# Investigating unexplained fatigue in general practice with a particular focus on CFS/ME

**DOI:** 10.1186/s12875-016-0493-0

**Published:** 2016-07-19

**Authors:** Amolak S Bansal

**Affiliations:** Department of Immunology and Allergy, St. Helier Hospital, Carshalton, Surrey SM5 1AA UK; The Sutton CFS Service, Sutton Hospital, Cotswold Rd, Sutton, SM2 5NF UK

**Keywords:** Chronic fatigue syndrome, Myalgic encephalomyelitis, CFS/ME, Medically unexplained fatigue, Diagnostic criteria, Scoring system, Differential diagnosis

## Abstract

Unexplained fatigue is not infrequent in the community. It presents a number of challenges to the primary care physician and particularly if the clinical examination and routine investigations are normal. However, while fatigue is a feature of many common illnesses, it is the main problem in Chronic Fatigue Syndrome/Myalgic Encephalomyelitis (CFS/ME). This is a poorly understood condition that is accompanied by several additional symptoms which suggest a subtle multisystem dysfunction. Not infrequently it is complicated by sleep disturbance and alterations in attention, memory and mood.

Specialised services for the diagnosis and management of CFS/ME are markedly deficient in the UK and indeed in virtually all countries around the world. However, unexplained fatigue and CFS/ME may be confidently diagnosed on the basis of specific clinical criteria combined with the normality of routine blood tests. The latter include those that assess inflammation, autoimmunity, endocrine dysfunction and gluten sensitivity. Early diagnosis and intervention in general practice will do much to reduce patient anxiety, encourage improvement and prevent expensive unnecessary investigations.

There is presently an on-going debate as to the precise criteria that best confirms CFS/ME to the exclusion of other medical and psychiatric/psychological causes of chronic fatigue. There is also some disagreement as to best means of investigating and managing this very challenging condition. Uncertainty here can contribute to patient stress which in some individuals can perpetuate and aggravate symptoms. A simple clinical scoring system and a short list of routine investigations should help discriminate CFS/ME from other causes of continued fatigue.

## Background

Tiredness is universally understood as an uncomfortable and sometimes disabling symptom that can reduce or prevent physical and mental activity. However, there is little knowledge of its precise nature and cause at a physiological and biochemical level. Chronic or persistent fatigue is under recognised and was found in 30.5 % of 9035 randomly selected adults in the Netherlands interviewed by questionnaire [1]. Importantly fatigue is a significant cause of impaired quality of life in a diverse range of illnesses including those with cardiorespiratory dysfunction, endocrinopathy, neurological impairment and neoplasia. While many illnesses are accompanied by fatigue, in only a few is it the main and most disabling symptom. In Chronic Fatigue Syndrome/Myalgic Encephalomyelitis (CFS/ME) fatigue is the predominant and most disabling symptom. It is frequently accompanied by several other symptoms that suggest a mixture of inflammatory, immune, viral and endocrine dysfunction [2–4].

Much has been written about CFS/ME in terms of physiology and psychology. There are conflicting views on its aetiology, diagnosis and management. Notwithstanding it is a relatively common condition with disabling fatigue as its primary problem [5] and presently waiting a precise and universally accepted patho-physiologic explanation. It is generally known that specialised services for CFS/ME are insufficient in coping with the demand and sometimes inadequate in the range of therapies offered. Confidently diagnosing CFS/ME as the cause of persistent fatigue can help allay patient and doctor anxiety, reduce the need for expensive investigations, allow early intervention and encourage higher rates of improvement.

### Historical

George Beard introduced the term ***neurasthenia*** in 1869 to describe an illness with chronic fatigue as its main symptom. This became a ‘respectable’ diagnosis from 1910 until 1980. There are a large number of conditions previously meeting many of the criteria for CFS and preceded in some cases by specific etiologic agents. These include myalgic encephalomyelitis (ME), post viral fatigue syndrome (PVFS), chronic fatigue and immune dysfunction (CFIDS), post-infectious fatigue syndrome (PIFS), fibrositis and myalgia.

### Prevalence of fatigue

Studies of the general population suggest a prevalence rate for CFS of between 0.2 and 2.6 % depending on the criteria used [5, 6]. More recent work suggests a figure of 1 % in the Netherlands [1]. The institute of medicine (IOM) in the USA has recently estimated that between 836,000 and 2.5 million Americans have CFS/ME [7]. This gives a prevalence rate of between 0.26 to 0.78 %. Most of the research on prognosis and treatment outcome has focussed on people attending specialist centres, who may be assumed to have more severe and complex difficulties. Nevertheless, studies suggest that a significant proportion of people with CFS/ME will continue to experience symptoms for some time [5]. Indeed, fewer than 5–6 % of people with CFS/ME return to pre-morbid levels of functioning in the medium to long term [6, 8]. The prognosis in children is significantly better with 80 % returning to normal health or much improved with mild persisting disability [9]. Improvement has been reported to be improved with intervention utilising a multidisciplinary treatment programme [10].

### The cost of unexplained fatigue and CFS/ME

Medically unexplained persistent fatigue causes considerable stress and is expensive for both the patient personally and for the UK National Health Service (NHS). A significant number of patients in this category undergo a vast array of expensive laboratory, radiological and other investigations that help to ‘exclude’ unusual and rare causes of persistent fatigue. In my personal experience I have seen several patients who have undergone whole body Magnetic Resonance Imaging (MRI) scans, echocardiography, tests of mitochondrial function, nutrient analysis, complex tests of immune dysfunction and rare infections totalling well over £10,000. As indicated by Mechanic [11] over two decades ago it can be difficult for doctors to know how far to pursue an underlying illness in a patient with ongoing and sometimes progressive symptoms. Quite often the cost equation is based on a mixture of the patient’s and doctor’s acceptance of uncertainty, the intellectual curiosity of the physician and the financial constraints of the individual and the healthcare system.

From the perspective of the CFS/ME sufferer the illness has a major impact on their quality of life [12], employment and in consequence household finance. Indeed approximately 50 % have had to cease employment as a result of their fatigue and other symptoms [13]. In the case of parents with children with CFS/ME, there was not only a net monthly loss of £247 per household but also increased expenditure of a similar amount [14]. Additionally, nearly three quarters of mothers of children with CFS/ME had a significantly impaired psychological health as assessed by the general health questionnaire-12 [14]. In the USA, Jason et al. [15] determined that CFS/ME cost the economy £9.1 billion based on ‘37 % decline in household productivity and a 54 % reduction in labor force productivity’. A more recent economic calculation by the Institute of Medicine (IOM) based on an analysis of the relevant literature suggested figures of between $17 billion to $24 billion.

### Diagnosing CFS/ME based on recognised criteria

Disabling fatigue that is sufficient to impair work, school and leisure activities and present for 6 months in adults and 3 months in children (in the UK the figures are over 4 months for adults and 3 months in children) is the hallmark of CFS/ME. However, it is hoped that diagnosis and therapy is offered as early as possible and especially in children. Nevertheless, all CFS/ME criteria were originally devised to standardise the diagnosis for research purposes. As happens frequently with many research criteria they have over time formed the basis for routine clinical diagnosis and with varying degrees of success. One of the earliest of the CFS/ME criteria included that formulated by Holmes et al. in 1988 [16] from the US Centre for Disease Control (CDC). These were drafted to help standardise the patient population for research purposes and to avoid the connection with viral infection after investigations failed to confirm past or current infections. The 1994 revision of the CDC case definition by Fukuda et al. [17] remained the main diagnostic CFS/ME criteria for several years and was used for patient selection in numerous research reports. However, the centrality of the delayed post exertion malaise that characterises CFS was lacking and in 2003 Carruthers proposed the Canadian criteria [18]. More recently the international ME criteria [19] have been drafted and both this and the Canadian criteria placed greater emphasis on the exacerbation of physical and mental symptoms by over activity. Unfortunately both sets of criteria are cumbersome and not easily adapted for routine use and particularly in primary care. The most recent criteria for CFS/ME have been proposed by the IOM [7]. These are simpler to use than previous suggestions but unfortunately do not stringently exclude psychiatric illness. Furthermore, it also appears to select patients with lesser degrees of functional disability and with fewer symptoms [20].

In the absence of diagnostic symptoms, signs and laboratory abnormalities, the confirmation of CFS/ME as the cause of persistent fatigue is presently based on the exclusion of an underlying medical and psychiatric illness known to be associated with fatigue. However, many primary care physicians have had no undergraduate or postgraduate training in the diagnosis of CFS/ME [21, 22] nor indeed in its management. In a recent report from a tertiary CFS/ME centre, confirmation of CFS/ME as a cause of persistent disabling fatigue was only evident in 23.3 % of the 279 patients studied. In 21.1 %, the CFS was accompanied by a sleep disorder and/or psychiatric disorder ‘…not invalidating the diagnosis of CFS’. In the remaining patients, 9.7 % had a predominant sleep disorder, 19.0 % had a psychiatric disorder and 20.8 % a combination of both. In only 2.2 % was ‘a classical internal disease’ evident [23].

With the above facts in mind and with certain knowledge about limited financial and manpower resources, I examined in 2008 the most frequent symptoms suffered by those with CFS/ME. These were weighted according to their importance in diagnosing the condition and to the exclusion of other causes of prolonged unexplained fatigue. The hospital and anxiety depression scale [24] was used to check for significant associated psychological dysfunction and a panel of basic blood investigations used to check for inflammation, autoimmunity, endocrinopathy and organ dysfunction. Patients with CFS/ME diagnosed in this way have been followed in the clinic for up to 7 years and an alternative explanation for the fatigue has been not been forthcoming in any patient.

### Symptoms and signs noted most frequently in CFS/ME

In addition to fatigue, individuals with CFS report a variety of other symptoms. These include musculoskeletal pain, sleep disturbance, impairment in short term memory and concentration, sore throat, and headaches of new type, pattern and severity [5–7]. In nearly all cases there is a delayed exacerbation of these symptoms, but particularly the fatigue, by any form of mental or physical exertion. This often leads to withdrawal from a wide range of activities. Symptom severity frequently, and often unpredictably, fluctuates on a daily or weekly basis. This often makes it difficult for patients to plan activities. The unpredictability of set backs contributes significantly to low mood and anxiety which in turn often perpetuates and aggravates CFS symptoms.

The frequency of symptoms reported by CFS/ME patients seen in the Sutton CFS service are detailed in Table 1. The results are based on the more than two thousand two hundred patients seen here between 2008 and 2015. When significant, intrusive and present for over 50 % of the time these symptoms are used in helping in the diagnosis of CFS and are included in many of the recent CFS/ME criteria. In contrast there are few signs that are easily discerned on the casual examination. Noteworthy, however, are the presence of an increased respiratory rate with intermittent bouts of sighing, and coldness of the extremities in those with moderate or worse CFS/ME. The reaction of the pupils to direct and prolonged illumination is unusual and the precise mechanism is unclear. These are discussed below.

**Table 1:** Symptoms and signs most frequently noted in those with CFS/ME

Symptoms	Signs
Aching muscles, 90 %+,	Pharyngitis, 25 %
Non-Restorative sleep, 95 %+	Cervical tenderness with or without lymphadenopathy 25 %
Daytime nap 3+ times per week, 30 %	
Muscle weakness, 90 %+	Axillary lymph nodes 10 %
Impaired concentration, 90 %	Cold peripheries in moderate, severe and very severe ME −70 + %
Forgetfulness, 85 %	
Muddled thinking, 80 %+	
Aching joints, 85 %+	Temperature >37.5C but <39.0C, 10 %
Stress aggravated Fatigue, 90 %	Increased respiratory rate, 80 + %
Headaches 75 %	Altered pupil reflexes, 60 %
Weight gain, 50 %	
Orthostatic intolerance 60 % [according to the IOM – [7]	

### Adapting presently recognised CFS/ME criteria for use in primary care

After long careful scrutiny of the various CFS/ME criteria and collation of the most important symptoms suffered by those with CFS/ME, I developed a scoring system to help colleagues to diagnose this condition in people presenting with fatigue. This is detailed in Table 2 and guidance on the frequency and severity of each symptom is detailed subsequently. As suggested by Jason et al. [25] a minimum frequency of 50 % and least moderate severity of each symptom is critical in helping to confirm CFS/ME as the mere presence of each may be evident even in a third of healthy controls.

**Table 2:** Details of the important symptoms that characterise CFS/ME and their respective scores. See text for further details

Factor	Score
Delayed prolonged post-exertion malaise after increases in physical, mental & emotional activity	3
Non-restorative sleep with frequent difficulty initiating and/or maintaining sleep	2
Impaired concentration that is reduced further by external stimuli	1
Reduced short term memory with word finding difficulty	1
New onset headaches (>2/mth and different in character from previous headaches)	1
Sore throat with cervical tenderness/recurrent flu-like episodes	1
Arthralgia affecting several joints with stiffness >1 hr but no swelling	1
Myalgia affecting multiple groups and exacerbated by mild exertion	1
Postural instability feeling unstable on standing, prolonged standing or sitting	1
Hypersensitivity to sounds and lights (smells and to a lesser degree taste also)	1

Unfortunately there is no gold standard for diagnosing CFS/ME and certainly there are no specific laboratory or radiological tests available. Nevertheless, the scoring system has now been used in the Sutton CFS/ME service for the last 7 years and assessed in well over two thousand consecutively seen patients. When used in conjunction with the baseline investigations listed in Table 3, it has proven to be remarkably robust when used by a range of healthcare personnel including physicians, nurses and physiotherapists. Indeed the scoring system has positively diagnosed CFS/ME to the exclusion of other causes in even quite complicated cases.

**Table 3:** Basic investigations in patients with prolonged fatigue of unknown cause

Investigation	Comments
FBC	Anaemia, polycythaemia, haematological malignancy can all be associated with fatigue. Red cell MCV may indicate need to check ferritin if reduced and vitamin B12 and folate deficiency if raised.
ESR	This is a good test of overall immune activation and a raised level should encourage an assessment of infection, autoimmunity, certain solid organ neoplasms and possible lymphoproliferation.
CRP	A raised level suggests inflammation somewhere. Where the source of the inflammation is not obvious from the history consider the sinuses, urinary tract
Urea, creatinine, electrolytes and Liver Function tests	Dysfunction in both areas can be accompanied by fatigue. There is an interesting association between Gilberts disease and fatigue [81] The mechanism is unknown.
Thyroid function tests	Both hypo and hyper-thyroidism can be accompanied by fatigue. In a small proportion of patients with anti-thyroid peroxidase antibodies but essentially normal T4 and TSH, low dose thyroxine can be helpful.
Autoimmune profile on tissue block	Can help check for Sjogren’s syndrome, early primary biliary cirrhosis and autoimmune hepatitis and atropic gastitris. The latter can be associated with vitamin B12 deficiency. A positive ANA here may encourage further tests of autoimmunity.
Anti-Tissue Transglutaminase (TTg) or endomysial antibodies	Coeliac disease can present with fatigue and without bowel symptoms.
Immunoglobulins and serum protein electrophoresis	Serum immunoglobulins are low in antibody deficiency but raised in chronic inflammation/infection. Both conditions can be accompanied by marked fatigue
Urine dipstick analysis	Simple check for renal inflammation/infection and renal tumours.

### Using the CFS/ME scoring criteria

The diagnosis of CFS/ME in adults is based on the presence of variably disabling fatigue of at least 4 months duration (6 months outside of the UK) and 8 or more from 13 points awarded for each of the symptoms and factors detailed in Table 2. However, there is an absolute requirement for some form of post-exertion malaise (PEM) after physical, mental and emotional over activity. To attract the maximum 3 points the PEM must be delayed by at least 12 h after the completion of the specific activity and the extra fatigue and additional ill health must be prolonged with a duration exceeding 24 h. Note that in children the PEM can be immediate and particularly in the very young children. If the PEM is more immediate and certainly less than 3 h after extra activity but the duration is still over 24 h then 2 points are awarded. A rapid PEM (<1 h) lasting less than 24 h gives 1 point. Alternative causes of the fatigue must be considered if it occurs simultaneously and commensurately with the physical over activity and particularly if there are any cardiorespiratory symptoms.

Unrefreshing sleep is a frequently reported by both young and old patients with CFS [26–28] and a disturbance of sleep has been demonstrated on polysomnography by several investigators [29–32]. Many CFS subjects also suffer difficulty with sleep initiation and maintaining sleep [29, 32]. A demonstrable reduction in the percentage of stage 4 sleep was evident in these patients on polysomnography [29]. Non-restorative sleep (NRS) affects over 90 % of patients with CFS/ME and often occurs in most, if not every, night. It is important that obstructive sleep apnoea, frequent waking from pain and urinary symptoms are excluded as a cause of NRS. Sleep studies are recommended if there are concerns as to the importance of sleep disturbance in those with chronic fatigue. This is important as recent work suggests that a primary sleep disturbance may explain fatigue in almost a fifth of patients with Fukuda criteria confirmed CFS/ME and is a comorbid problem in a similar proportion [23]. Two points are given if the NRS is accompanied by difficulty initiating and maintaining the sleep at night time and these difficulties are evident for 5 or more nights per week. However, if the NRS is not accompanied by difficulty initiating or staying asleep this gives 1 point. In both cases the non-restorative nature of the sleep disturbance must persist with hypnotics which many patients use to initiate sleep.

Regarding, the neurocognitive problems faced by those with CFS, the two that appear to be most disabling are the impaired concentration and short term memory. These are aggravated by extraneous noise, bright lights and movement. Word finding difficulty is especially frequent and ‘following’ a conversation is often mentioned as being difficult especially one involving an unfamiliar topic. Both the reduced concentration and the impaired memory are given one point each although it may be argued that the two are related. Thus concentration is required to acquire memory and the latter may be reduced because of problems with the former. Notwithstanding, we have recently shown that deficits in recall are more significant for retrospective than prospective memory and that fatigue, depression and general efficacy were most linked with cognitive failures [33].

The aching of the muscles of the axial and appendicular skeleton has a quality similar to that experienced when an individual has a viral infection. The muscle creatine kinase is normal and while there is no clear evidence of muscle damage, some have reported mitochondrial abnormalities [34] and virus like particles. In the absence of fibromyalgia there is no significant and painful tenderness of the muscles. The latter can be seen in polymyositis which like many of the systemic connective tissue disorders can be accompanied by fatigue in a significant proportion of patients [35, 36]. The arthralgia in CFS/ME is rarely accompanied by joint swelling and there is no synovitis. The majority of patients mention stiffness of the joints which can last over one hour in the morning. Both the myalgia and the arthralgia give one point each only if these symptoms are evident for more than half of the patient’s waking hours and the myalgia is aggravated by low level activity.

The mechanism of the hypersensitivity to lights and sounds and sometimes smells is unusual and this symptom is rarely seen in any other condition. In this respect some patients with significant CFS/ME may chose to wear sunglasses even in normal daylight. For some the hypersensitivity to smells contributes to their continual nausea. It is unclear whether the myalgia and arthralgia reflect a general hypersensitivity to all sensory stimuli but both need to be present for more than half of the waking time to attract one point each. Many CFS/ME patients report that their joint pain is accompanied by stiffness which lasts for hours and may be present all the time. The joint stiffness in rheumatoid arthritis and the recognised connective tissue disorders is nearly always worse after rest and inactivity and in the morning tends to last only a couple of hours or so. In these conditions synovitis with swelling and warmth of the joints is often seen and particularly with acute flares of disease activity. In contrast joint swelling and warmth was rarely, if ever, seen in any of our CFS/ME patients. Where there is doubt, blood tests looking for inflammation, immune activation and autoimmunity can be very helpful.

Sore throat and flu like sensations have been suggested to support the notion that CFS/ME is due to an unrecognised viral infection that activates recurrently. However, throat swabs rarely prove positive for either bacteria or the commonly recognised viruses. Regardless, the sore throat is frequently associated with tenderness of the anterior and sometimes posterior neck although actual lymphadenopathy is frequently absent. To score a point this symptom must be evident one or more day per week and for at least the previous four months. In reference to the flu like sensations these often cause aching of the body and with a low grade fever or sensation of being hot and sweaty. In my experience these can last several hours at a time and occur at least once per week in over two thirds of patients.

The presence of eight or more points out of thirteen gives a high probability that the patient has CFS/ME. For research studies I would suggest that subjects should have ten or more. This is the case for the research undertaken at this institution. In borderline cases where the subject has prolonged unexplained fatigue but scores only 7 points, the presence of certain other unusual features may be used to confirm the diagnosis of CFS/ME. These include alcohol intolerance, hypersensitivity to medications, perpetually cold hands and feet and an unusual recurrent sighing pattern of respiration.

### Assessing chronically fatigued patients with borderline scores

Additional features that may suggest CFS/ME in those with borderline scores include alcohol intolerance which we see in four fifths of our patients. This often commences soon after the onset of the CFS/ME symptoms. In the only other report on this issue, Wooley et al. [37] reported 67 % of their 114 patients with CFS reducing their intake of alcohol as it made their fatigue worse. In our experience nearly all patients with CFS have reduced their intake of alcohol and I have not seen a single patient who had increased his/her alcohol intake. Moreover, tolerance of 4 units or more of alcohol in a single sitting is unusual and encourages us to revaluate the diagnosis. In this regard fewer than 20 % our patients continue regular alcohol ingestion although even here the amount consumed has been decreased.

In regard to a hypersensitivity to drugs this is especially evident to serotonin specific reuptake inhibitors (SSRIs), serotonin and noradrenaline reuptake inhibitors (SNRIs) and tricyclic antidepressants (TCAs) and we see this in at least half of our patients. The hypersensitivity leads many with CFS/ME offered this type of treatment to cease the medication within the first week of commencement. Often this is due to marked imbalance, state of dysreality and general increase in the fatigue and other CFS/ME symptoms. I would suggest that patients with CFS/ME who have developed depression should be commenced at one quarter of the lowest starting dose for a week. This is doubled over the next 2 weeks and subsequent increases are undertaken according to the patient’s response.

Many people with CFS/ME mention that their hands and feet are perpetually cold. This is often evident on shaking the patient’s hand and is seen in over three quarters of patients with moderate or severe CFS/ME. In those with mild CFS/ME, cold peripheries are evident less frequently and in my experience probably a quarter or less of the patients will be aware of this problem. In a small proportion of CFS/ME patients features highly suggestive of acrocyanosis are evident. In my estimation this is less than 5 % although it is interesting that acrocyanosis may be present in 50 % in those with the postural orthostatic tachycardia syndrome (POTS) [38]. Regardless, the mechanism of the cold peripheries is unclear but may relate to persistently high adrenaline/noradrenaline levels causing peripheral vasoconstriction with a view to maintaining central blood pressure and perfusion of the vital organs. A similar mechanism as well as possible primary hypovolumaemia and adrenaline receptor autoimmunity is proposed in patients with POTS [38] and the latter is especially frequent in CFS/ME [39, 40]. More frequent still is orthostatic intolerance suggested to affect over 60 % of CFS/ME patients in the IOM report [7]. The overlap between CFS/ME and POTS is intriguing as both have fatigue, disturbed sleep and impaired mental processing as frequent symptoms. Further work is clearly required to determine precise differences and overlap between these conditions which may influence and direct specific treatment strategies. Equally important would be investigations into the value of measuring orthostatic intolerance in the clinic as a test aiding the diagnosis of CFS/ME.

The respiratory rate in patients with CFS/ME has been infrequently studied. However, hypocapnia has been described in CFS both at rest and on tilt table testing [41]. However, Saisch et al. [42] found only a weak association between hyperventilation and fatigue symptoms in 30 patients diagnosed with CFS on the basis of the Oxford and CDC criteria. Regardless, clinical symptoms suggestive of hyperventilation are seen in about of quarter of our patients but many more have an increased respiratory rate. Indeed in the experience of this unit we see an increased respiratory rate in approximately 80 %. This is only slightly less than the 93 % observed in the 100 patients with ME or post-viral fatigue investigated by Rosen et al. [43]. Interestingly, efforts to reduce the tendency to hyperventilation using breathing control exercises have been found helpful in about a third of our patients in terms of reducing fatigue, impaired concentration and memory and brain fog. These breathing control exercises include the Butekyo method, mindfulness meditation and abdominal breathing exercises. In a smaller proportion of cases the regulation of fast breathing has improved episodes of dysreality that were suggestive of hypocapnia.

Abnormalities of the pupils to light are seen in patients with cerebral ischaemic events, certain brain neoplasms, local disturbance in neural transmission and demyelination. Checking the pupil reflexes is often undertaken to detect alterations in the brainstem pathways mediating the perception of light, accommodation to distance and near vision and changes based on ambient illumination. In patients with CFS/ME I have observed two unusual responses that are evident on prolonged illumination of the pupils. The more frequent finding seen in three quarters of patients is a rhythmic contraction and dilatation of the pupils. The second pattern is a paradoxical dilation of the pupils after an initial contraction. The latter is seen in patients with autoimmune autonomic neuropathy that is caused by IgG antibodies to the ganglionic acetylcholine receptor [44]. However, these auto-antibodies are absent in the patients with CFS with this type of pupil abnormality. Interestingly these CFS/ME patients are more frequently faint on standing, have poor tolerance of prolonged standing and other features of POTS. In my experience other symptoms of a more generalised autonomic neuropathy are absent. The cause of these changes in the pupil reflexes is unclear but a persistent state of adrenergic over activity may be involved. This may oppose the constricting action of direct pupil illumination and produce the fluctuating response in many and a more significant but delayed dilatation in others. Interestingly, a delay in pupil constriction to direct light was reported in depressed patients by Fountoulakis et al. [45] and attributed to reduced noradrenergic tone.

From a clinical perspective, the above five features of CFS/ME may help in confirming the diagnosis when patients have borderline main criteria or where there is doubt for other reasons. In this case the presence of three or more of these additional points of cold peripheries, alcohol intolerance, drug hypersensitivity, altered pupil reflexes and altered respiration gives one extra point.

### Investigating unexplained fatigue

It cannot be emphasized enough that the most important part of the diagnostic process in investigating unexplained prolonged fatigue and suspected CFS/ME is a full and careful history and examination. The aforementioned scoring system appears in our hands to be especially useful in diagnosing CFS/ME and distinguishing it from other causes of prolonged fatigue. Unfortunately there is presently no one diagnostic test for CFS/ME. However, a few basic and readily available tests are important to check for the presence of inflammation, immune activation, essential organ dysfunction, endocrine abnormalities, gluten sensitivity and autoimmunity. These tests are detailed in Table 3 and negative/normal results make other causes of the fatigue very unlikely. Performed within NHS laboratories the total cost of these tests in 2015 is less than £80.

Additional tests may be needed in a small proportion of people and these are guided by the presence of specific symptoms. Other tests are guided by a history of specific exposures to infectious agents such as to ticks which can be associated with Lyme disease. The list here is not exhaustive and further detailed tests may needed and again these are guided by specific patterns of muscle disease, sensory abnormalities, joint problems or endocrine abnormalities. Given the absence of universally acknowledged effective treatment that restores the person back to premorbid health, I would not presently recommend expensive tests of immune and muscle function, nutrient analysis and in depth brain imaging. These tests are best performed in specific research projects and include the enumeration of NK cell numbers, assessment of NK cell cytotoxicity, T and B cell subset analysis and checks of serum/plasma Th1/Th2/Tregs/Th17 associated cytokines. For the same reasons I think it is presently unwise to assess RNAse L levels and perforin activity as there are no easily administered low cost low toxicity treatments presently available. Tests of mitochondrial function are also available but it is presently unclear how these are altered in such a marked and diverse way by inactivity alone. Additionally, the treatments recommended to correct mitochondrial deficiencies are nutritional supplements that that are readily available at modest cost and with virtually no side effects when taken according at recommended daily allowance (RDA). As such a simple trial of these therapies for two or three months may be safely undertaken and without the need for the expensive tests. In my experience the use of these supplements may partially help some of the symptoms of CFS in a small group of patients for a variable period of time but no one has been cured.

After full clinical history and examination the tests detailed in Table 4 may be considered. Several points in the patient’s clinical history may be relevant in tailoring subsequent therapy but are not used in making or supporting the diagnosis of CFS/ME. These include significant negative life events at the time of onset of CFS/ME symptoms. Indeed stress at the time of a triggering infection or event is reported by over half of the patients seen in this service. It is possible that this may impair cellular immune functioning that allows greater viral dissemination and reduced clearance that perpetuates the fatigue and other symptoms [46]. In these individuals the onset of the CFS is relatively acute and symptoms noted over a period of several days or a few weeks. In others the onset is gradual over many weeks and sometimes months but on-going stress does appear to be evident in the most of the patients. Physical Injury precipitating CFS appears to be rare although there is some evidence that it can lead to fibromyalgia. In contrast CFS/ME occurring after an operation is seen occasionally and perhaps one in fifty patients. Whether the anticipation and the associated stress are important is unclear. Environmental toxins have long been cited as contributing to CFS/ME and organophosphate pesticides as well as multiple vaccinations in the gulf war syndrome are two notable examples [47].

**Table 4:** Further investigations in patients with chronic fatigue of unknown cause

Clinical Symptoms/Signs	Additional investigations
Evidence of Connective Tissue Disease suggested by Raynaud’s phenomenon, mouth ulcers, photosensitive rash, serositis, synovitis,	Anti-nuclear antibody assessment on Hep2 cells, antibodies to ENA and dsDNA. Rheumatoid factor analysis and anti-CCP antibodies
Muscle tenderness or history of significant exercise related cramps	Creatine Kinase, Lactic dehydrogenase, Liver function tests. Consider EMG and possibly muscle biopsy. Referral for late presenting inherited muscle or glycogen storage diseases.
Widespread aches and pains especially in older women	Serum calcium and magnesium estimation and DEXA scan for osteoporosis and hyperparathyroidism. Serum immunoglobulin assessment in basic panel will check for myeloma.
Addison’s/Cushings disease	Synacthen/Dexamethasone suppression test. Random cortisol levels can be reduced in CFS/ME and synacthen is advised. Cortisol awakening response is blunted in CFS/ME and might relate to the exacerbation of fatigue in the morning with difficulty getting up in some patients.
Tick bites with erythematous rashes and arthralgia and fatigue	Serology for Lyme disease – care with interpretation of results and particularly with results from non-approved laboratories. Lyme disease is rare in the UK especially in areas without deer populations.
Neurological abnormalities, reduced mental acuity and progressive confusion, leg weakness and bladder/bowel problems	MRI/CTscan of brain for cerebral atrophy, ischaemic areas, plaques of demyelination, tumours in frontal lobes/para-saggital area and possible Arnold Chiara malformation. Consider also neuropsychological testing.
Intolerance of prolonged standing, recurrent syncope/presyncope, tachycardia within 10 min of standing or marked hypotension on standing with tachycardia.	Tilt table testing for autonomic dysfunction and further evaluation for postural orthostatic tachycardia
‘Clicky’ joints with previous dislocation(s), early stretch marks and easy bruising	Consider referral for formal evaluation of an underlying or complicating joint hypermobility syndrome.
Significant sleep disturbance	Sleep studies. Frequent sleep arousals can cause marked daytime fatigue.

A family history of CFS/ME and certain personality traits are also noted to be increased in those with CFS/ME including conscientiousness and high personal and parental expectations [48]. Ciccone et al. [49] while noting a higher prevalence of psychiatric illness and personality disorder in CFS/ME did not find any major correlation between these and levels of physical functioning and disability. More recently we have noted an increased frequency of CFS/ME symptoms in those with borderline personality disorder which has not hitherto been reported. Previous work has also suggested an increased prevalence of childhood physical, emotional and sexual abuse in those with idiopathic chronic fatigue and chronic fatigue associated with medical and psychiatric illness [50]. Borsnini et al. [51] have also confirmed childhood stressors to increase the possibility of subsequent CFS/ME. We do not routinely explore this area in detail as it seldom helps to reduce the symptoms of CFS/ME but does have the possibility of aggravating matters by raising stress and anxiety levels. In some instances where the past impacts significantly on the present, referral to an appropriate psychologist may be helpful. However, patients are warned that there may be an initial deterioration in symptoms before any improvement.

### Important considerations in the differential diagnosis of CFS/ME

While virtually all illness is accompanied by at least some fatigue, this is the major problem in those with CFS/ME. The illnesses that have significant levels of fatigue as the main symptom and which may pose greatest difficulty in diagnosis are detailed in Table 5. For many there are laboratory and/or radiological tests available that may establish the diagnosis once the condition has been considered. How joint hypermobility causes significant fatigue is unclear but chronic fatigue was observed in 82 % of 466 adults with self reported JHS assessed by Murray et al. [52] in an extended on-line questionnaire. Interestingly persistent anxiety and depression were also very frequent in this group of individuals [52]. In children, JHS has been reported to be much more frequent in those with CFS/ME and with an odds ratio of 3.5 [53]. In my experience JHS is present in approximately 20 % of patients with CFS/ME and the majority will have Ehlers Danlos Syndrome joint hypermobility type. Interestingly, patients with JHS had a significantly higher frequency of orthostatic intolerance than healthy controls [54]. In patients with Ehlers Danlos syndrome joint hypermobility type, fatigue is especially frequent and appears related to the degree of orthostatic intolerance [55]. The present CFS/ME definitions do not consider Ehlers Danlos Syndrome joint hypermobility or JHS as exclusionary conditions. However, the overlap between these conditions and CFS/ME is significant and appears to be underestimated. Future research in CFS/ME needs to separate these groups to see if distinct patho-physiological mechanisms are operative.

**Table 5:** Differential diagnosis in patients with prolonged fatigue of unknown cause

Easily missed conditions that may cause unexplained fatigue	Comments
Ehlers Danlos Syndrome type 3 - Joint hypermobility type	Unclear how this predisposes to chronic fatigue and CFS/ME but muscle strengthening around joints and the back can be helpful but not curative.
Hypothyroidism, Addison’s disease and Pituitary dysfunction	Care with post traumatic head injury leading to pituitary dysfunction – check for significant head injury even years beforehand. Consider synacthen and glucagon stimulation tests.
Sjogren’s syndrome, early PBC, other CTD	Note that dry eyes and mouth without overt Sjogren’s syndrome can be seen in CFS/ME. The autoimmune profile, serum immunoglobulin assessment and ESR should help check for these possibilities.
Coeliac disease	People with anti-TTg antibodies but without overt celiac disease evident on duodenal biopsy can sometimes see an improvement in their fatigue on gluten avoidance
Generalised anxiety disorder (GAD) and depression	Important to note that anxiety and depression can complicate CFS/ME and treatment for these can help fatigue overall.
Primary disorders of sleep	While obstructive sleep apnea can be associated with fatigue and day time sleeping, frequent sleep arousals without significant episodes of apnea can also lead to persistent daytime fatigue.
Early dementia, multiple sclerosis and Parkinson’s disease	MS can be associated with marked fatigue, however, the twitching, sensory symptoms and blurring in CFS/ME are brief lasting less than a couple of hours while those in MS last days and weeks. Diagnosing CFS/ME in the elderly is more difficult and several neurological conditions can cause marked fatigue. Fatigue is frequent in early Parkinson’s disease.
Postural orthostatic tachycardia (POTS)	This may be a primary condition with associated fatigue but without the other symptoms of CFS/ME. However POTS is also not uncommon in those with moderate and severe CFS/ME as is vaso-vagal syncope.
*Other conditions*	*There are several conditions in this category that include cardiac dysfunction, temporomandibular disorder but which are very rare.*

Early dementia can rarely present with chronic fatigue and the diagnosis can be very difficult. Two out of the two thousand patients seen in this service had early dementia presenting with fatigue as the main problem. Neuropsychological testing can be helpful and cerebral imaging increasingly useful with disease progression. The Epworth sleep scale is an easily completed self rated questionnaire that can help in the investigation of sleep disturbance in chronic fatigue. A score of 16 or more should encourage a search for sleep factors that may be impacting on the chronic fatigue. This is especially helpful in those patients with significant snoring and high body mass index (BMI).

### CFS/ME and depression

It is commonly acknowledged that fatigue is commonly seen in patients with depression and indeed many other mental illnesses. However, there are several features that show clear difference between those with depression and those with CFS whose illness has not been complicated by depression and anxiety. These are summarised in Table 6. CFS/ME has been, and continues to be, considered by many as a somatisation disorder (see Lakhan and Schofield [56]). Two points have been used to support this assertion. Firstly the relatively high rate of past psychiatric illness in those with CFS/ME. Thus previous work using the older CFS/ME criteria suggested that two thirds of patients had features of a major depressive illness and one half of all patients with CFS had experienced at least one episode of major depression [57, 58]. More recently, Nater et al. [59] using a patient completed Structured Clinical Interview for DSM-IV reported almost nine tenths of CFS patients had one or more lifetime psychiatric diagnosis and over half had one current psychiatric diagnosis. Using the more recent Canadian and International ME criteria to confirm CFS/ME, the frequency of depression is much less [60] and these criteria do appear to select out those individuals in whom depression may be a significant factor in chronic fatigue.

**Table 6:** Clinical features that help to distinguish depression and CFS/ME

Variable	Depression	CFS/ME
Physical Exertion	Exercise can improve mood and energy levels overall	Nearly always causes delayed worsening of the fatigue and other symptoms
Mood	Low	Usually normal
Motivation	Reduced	Normal in the majority and in the absence of complicating depression
Sleep	Early morning wakening common but difficult initiating sleep also seen	Difficulty initiating sleep and getting up in the morning
Memory	Often rumination about the past and feelings of guilt	Word finding difficulty and precise recollection of recent events.
Concentration	If engaged can be normal	Impaired especially with extraneous noise and movement
Energy	Persistently low but with only minor day to day variability and no delayed post-exertion worsening	Variable from day to day and accompanied by delayed worsening with physical, mental and emotional exertion
Appetite	Low. Weight can go down.	Normal. Weight either maintained or sometimes increased.
Affect	One of sadness. Reduced spontaneity of action and of facial expression	Normal. Frustration and sometimes anger seen.
Interest in outside life	Reduced. No desire to complete previous hobbies or see family and friends	Maintained. Impaired energy reduces ability to continue with hobbies, social life and leisure activities.
Response to anti-depressants	Fatigue may be improved	Little or no response if no complicating depression and many are hypersensitive to normal starting doses.

The second issue concerns the high frequency of depression associated symptoms in those with CFS. As such the fatigue, non-refreshing sleep, bodily aches, impaired concentration and memory observed in depression has been used to support the notion that those with CFS must have depression if they do not have laboratory evidence of an organic illness. Interestingly the nature of fatigue in depression has not been well investigated and only recently has it been investigated in illnesses presently recognised to be the result of organic processes. These include particularly Sjogren’s syndrome, celiac disease, primary biliary cirrhosis and multiple sclerosis. Regardless, the present focus of treatment in CFS/ME in the UK remains centred on the correction of disordered thought processes in relation to fatigue. These include challenging unhelpful patterns of thought as part of CBT, encouraging the patient to do more as means of correcting muscle deconditioning, encouraging them to relax and reduce the ability of adrenaline to deplete mental energy reserves. Further therapies utilising tricyclic anti-depressants (TCA), serotonin specific reuptake inhibitors (SSRI) and serotonin and noradrenline reuptake inhibitors (SNRI) have the aim of increasing the patient’s sense of well being and reducing depression. Thus many consider the symptoms of CFS/ME to be primarily psychological in nature. However, significantly low mood is denied by the majority of patients with CFS and particularly by those who have recovered from depression and recognise its clinical features.

The majority of patients with CFS/ME are keen to deny that they have depression. Certainly their affect does not often suggest this as a major problem. However, as with all chronic illnesses, depression can set in if the disabling symptoms remain unexplained, there is unpredictability of relapses and there is no improvement despite the best efforts of the patient and his/her physicians. In this regard comorbid depression was noted in a fifth of the Fukada criteria diagnosed patients reported by Mariman et al. [23]. Three points are relevant in distinguishing depression and CFS/ME. Firstly diagnosing depression according to the DSM IV inherently includes fatigue, which is seen frequently in depression, but is the main symptom of concern in those with CFS/ME. Additionally, many of the physical symptoms seen in depression overlap with those observed frequently in CFS/ME. These include low energy, sleep disturbance, poor memory, impaired concentration and aching. The differences in the specific nature of these and other symptoms seen in these two conditions are detailed in Table 6. Secondly, reports assessing the frequency of depression and anxiety in those with CFS/ME have investigated patients when the diagnosis has been established. By definition this can only be at least four months after the onset of symptoms. By this stage many patients are saddened and perplexed by their illness and it is not surprising that their mood is low and some will be depressed. Nevertheless, many remain positive although frustrated and sometimes angry about the lack of understanding and sympathy shown by many in the caring professions. Thirdly, CFS/ME is more frequent in those with previous depression and there is a tendency to attribute new or on-going fatigue to a reactivation of depression rather than reassessing the specific nature of the fatigue.

There are few reports that have aimed to assess the presence of depression at the onset of CFS/ME symptoms. In those suffering an acute onset after a viral type illness, depressive symptoms have been absent although increased levels of work and life associated stress may have been evident. This is also the case in those whose illness started after physical trauma. In any case the presence of coincident depression later in the illness is often reactive to the patient’s continuing symptoms and disability. Regardless, its presence can exacerbate the individual’s symptoms, further impair quality of life and reduce the efficacy of both the talking and physical therapies. As such its early detection and appropriate treatment can undoubtedly be helpful.

As mentioned previously care is required in the use of anti-depressant medication in those with CFS/ME. Thus many agents introduced in conventional dosage induce a state of tension and irritability with a worsening of the fatigue and headaches, increased difficulty sleeping and sometimes muscle twitching. Indeed if these symptoms develop in patients with depression when commenced on SSRI, SNRI or TCAs then an underlying CFS/ME should be suspected and investigated. Although these symptoms can also be seen in people without CFS/ME it is certainly more frequent and prolonged in those with CFS/ME. The mechanism is unclear but a pre-existing state of central nervous system hyperactivity may be aggravated by these agents that often work by inhibiting the reuptake of serotonin and noradrenalin. In some ways the adverse response suffered by CFS/ME patients is similar to a mild serotonin syndrome or in some cases an anti-cholinergic syndrome. Regardless, anti-depressants should be introduced very slowly in those with CFS/ME and probably commencing at a quarter of the lowest strength medication. They should then be increased every two weeks until a therapeutic level is reached although quite often this remains at the lower end of that needed for those without CFS/ME. It should of course be noted that depression and anxiety can manifest many of the symptoms seen in CFS/ME. Additionally, depression and anxiety can complicate CFS/ME and prolonged depression and anxiety can be aggravated by CFS/ME. Separating the two can be difficult and the some of the more important distinguishing features are detailed in Table 6. However, for depression the pattern of sleep disturbance often confirms early morning wakening while in those with CFSME, patients may have difficulty getting up in the morning. Additionally, patients with CFS/ME say that they can start a task but have difficulty finishing it while those with depression find it difficult to initiate the task but once started can often finish it. Interestingly, some people with depression and anxiety resort to increased alcohol ingestion while those with CFS/ME almost never do.

### CFS/ME and anxiety

Anxiety is a frequent problem in patients with CFS/ME and may progress or be complicated by a generalized anxiety disorder, social phobia, panic disorder and agoraphobia [61]. This is understandable when the unpredictable onset and nature of relapses are considered. A generalised anxiety disorder was also noted to be more frequent in patients with CFS/ME than healthy controls and intriguingly had an early onset in a significant proportion [62]. Interestingly, the perception of stress has been shown to be increased in women with CFS/fibromyalgia compared to healthy women and in women with a medically accepted illness such as lupus, rheumatoid arthritis and multiple sclerosis [63]. This was associated with increased depression scores and more frequent unsupportive relationships. In my experience, persistent anxiety is a more significant problem in CFS/ME than depression and quite often more difficult to manage than the latter. CBT can be helpful to a degree but quite often the hypervigilance persists and sometimes the catastrophising also. Indeed Crawley et al. [64] in the UK, noted a reduction of only 0.6 (95 % CI -0.9 to −0.3) in anxiety assessed using the HADS at 8 to 20 months follow-up after treatment in 6 specialist CFS/ME services.

### CFS and Treatment

There is a large, and increasing, literature on a wide range of treatments used in CFS/ME. These include immunological and pharmacological treatments, nutritional supplements, adaptive pacing, graded exercise, deep relaxation using different approaches and psychological therapy [5, 6]. Included in the latter is cognitive behaviour therapy (CBT) and various types of life coaching. It would be impossible to cover the merits or otherwise of these treatments in this brief review. However, partly as a result of the unclear aetiology and heterogeneity of the condition, there are no CFS/ME treatments universally accepted by all physicians and patients to provide a permanent cure for the condition. Given that the precise cause of CFS is presently unknown, current treatment strategies should utilise an individualised holistic approach. This should include various combinations of pacing of daily physical and mental activity to prevent relapsing remitting fatigue, optimal diet, appropriate sleep hygiene, pharmacotherapy for any secondary depression/anxiety, graded exercise therapy (GET) and CBT.

The UK NICE guidelines on CFS/ME [65] have focused on GET and CBT in particular. Prinzs et al. [66] advocated the benefits of CBT which was also supported by the uncontrolled study of 125 CFS patients in the Netherlands by Scheeres et al. in 2008 [67] who found benefit in just over a third of the patients. Importantly, while cost outcome ratios were estimated to be highly favourable in each recovering patient, it is unclear whether they were favourable as a total cost of the intervention. The Cochrane review in 2008 also supported the use of CBT in CFS/ME although the benefits were modest and the duration of benefit was far from clear [68]. However, multidisciplinary input with CBT, GET and pharmacological treatment fared no better than exercise, counselling and pharmacological treatment in the Fukada criteria CFS/ME patients reported by Núñez et al. [69]. A year later the PACE trial [70] with much larger numbers of patients confirmed that both CBT and GET were statistically beneficial when compared to adaptive pacing and normal medical care. However, the overall improvement after one year of therapy and input was extremely modest and the number of people who managed to return to work was not reported.

In contrast to individualised CBT, group CBT investigated in the UK provided little improvement in fatigue [71]. Regarding other listening therapies, recent work has found little benefit from supportive listening and pragmatic rehabilitation delivered by nurses compared to treatment as usual offered by general practitioners [72]. Interestingly, multidisciplinary input has allowed a significant number of those with post-infectious CFS to return to work but was totally unsuccessful in those without an infectious onset [73]. Support groups and a positive physician-patient relationship have proven to be beneficial in the long-term management of CFS [74]. Overall, GET has been shown to be helpful in reducing fatigue and with a perception of improved sleep and general well being in those with CFS/ME diagnosed using a variety of criteria [75]. GET also showed little risk of harm to patients but does in my opinion need to be properly supervised by therapists knowledgeable about CFS/ME. The baseline from which the GET is commenced is particularly important as is the rate of increase in the chosen activity. In my opinion the input of a non-judgemental, caring and understanding primary care physician is extremely important in the long term management of those with CFS/ME. The early identification of CFS/ME and its management with firm reassurance, appropriate rest and activity may encourage improvement in a higher proportion than would otherwise be the case.

The present CFS/ME literature indicates that concomitant illnesses such as irritable bowel syndrome, fibromyalgia, headaches, and depression need to be treated and tailored to each patient to help significantly improve their quality of life. Psychopharmacologically, the use of SSRIs, SNRIs and TCA are suggested if depression complicates prolonged CFS/ME. In my experience there is no single agent that is superior to others and the precise agent used should be based on the physician’s experience and the patient’s tolerance to individual agents. Although clinical trials of tricyclic antidepressants have not produced definitive results, it is believed that along with their anti-depressive effect they also promote stage 4, non-rapid eye movement sleep and stimulate the descending inhibitory pathways of pain control. However, as with all medication these need to be introduced at the very lowest dosage and increased only gradually. Efforts at improving sleep and reducing insomnia have also been shown to not only reduce fatigue but also improve recovery from a stressful situation [76].

Many patients with CFS/ME experience chronic widespread pain. In many this responds poorly to conventional anti-inflammatory agents. The synthetic opiate agonists can be helpful in a small number of patients but have the propensity to induce dependence in some and can also contribute to disturbed attention and bowel disturbance. The gamma amino butyric acid agonists such as gabapentin or pregabalin can be more helpful. Low dose pregabalin can also provide benefit for mild associated anxiety. The headaches suffered by many with CFS/ME can be highly disabling. Not infrequently they have a multifactorial aetiology including an underlying migrainous tendency, cervical spine tension and stress related frontal tension. The migrainous component can be helped by propranolol in those without asthma and peripheral vascular issues and other helpful agents include pizotifen as well as topiramate. For insomnia, melatonin should be tried and in some patients the older anti-histamines such as promethazine and hydroxyzine can be helpful although daytime drowsiness may require careful titration of the dose. The newer non-benzodiazepine hypnotics such as zopiclone also have a place in managing insomnia in CFS/ME although I suggest that their use is restricted to perhaps 3 times week. For the orthostatic intolerance increased salt intake (5gm to 10gm per day) or the isotonic sports drinks can be tried. Fludrocortisone would present an alternative. For other agents such as ivabradine and midodrine referral to a cardiologist with an interest in POTS is suggested.

A lot has been written about dietary supplements and exclusion diets in CFS/ME [77, 78]. In my opinion a healthy well balanced diet with food that the patient enjoys eating is the best treatment for patients with CFS/ME. A small proportion of patients find wheat reduction helpful for reducing abdominal bloating and cramping and occasionally also increases energy levels. If dietary assessment suggests inadequate variety and paucity of specific types of food then dietary supplementation with a multivitamin and multimineral preparation may be appropriate. However, in the absence of any demonstrable nutritional deficiency the use of vitamin and mineral supplements is rarely required. Nevertheless, many patients still use a variety of dietary supplements and these are sometimes also suggested or prescribed by clinicians. Some patients have found benefit from the use of products that contain intermediates in oxidative cellular metabolism. These include factors such as co-enzyme Q, L-carnitine, D-ribose and nicotamide adenine dinucleotide (NAD). Regarding the latter Forsyth et al. [79], reported promising results with reduced NADH therapy that improved adenosine triphosphate levels. In my experience many of these compounds appear to provide only temporary benefit and few patients report consistent and definite benefit beyond a few weeks and occasionally months.

## Conclusions

In many ways it is unfortunate for sufferers with CFS/ME that it is a symptom based diagnosis without any easily discerned distinguishing physical or laboratory findings [5, 7]. There is, at present, no consistent pattern of immune dysregulation, endocrine dysfunction, nutritional deficiency, persistent infection or food allergy/hypersensitivity evident in this condition. It is likely that CFS/ME is a heterogeneous disorder [80] with multiple causes and exacerbating factors. Early diagnosis based on positive criteria reduces the need for expensive investigations and can reduce the prolonged stress that accompanies ill health symptoms without a diagnosis. Overall this may also allow an improved understanding of the condition and early intervention that may encourage improvement.
